# Multimodal MEMS vibration energy harvester with cascaded flexible and silicon beams for ultralow frequency response

**DOI:** 10.1038/s41378-023-00500-8

**Published:** 2023-03-23

**Authors:** Haizhao Feng, Ling Bu, Zhangshanhao Li, Sixing Xu, Bingmeng Hu, Minghao Xu, Siyao Jiang, Xiaohong Wang

**Affiliations:** 1 School of Integrated Circuit, Tsinghua University, 100084 Beijing, China; 2 School of Information Engineering, China University of Geosciences, 100083 Beijing, China; 3 College of Semiconductors (College of Integrated circuits), Hunan University, 430001 Changsha, China

**Keywords:** Electrical and electronic engineering, Structural properties, Nanoscale devices

## Abstract

Scavenged energy from ambient vibrations has become a promising energy supply for autonomous microsystems. However, restricted by device size, most MEMS vibration energy harvesters have much higher resonant frequencies than environmental vibrations, which reduces scavenged power and limits practical applicability. Herein, we propose a MEMS multimodal vibration energy harvester with specifically cascaded flexible PDMS and “zigzag” silicon beams to simultaneously lower the resonant frequency to the ultralow-frequency level and broaden the bandwidth. A two-stage architecture is designed, in which the primary subsystem consists of suspended PDMS beams characterized by a low Young’s modulus, and the secondary system consists of zigzag silicon beams. We also propose a PDMS lift-off process to fabricate the suspended flexible beams and the compatible microfabrication method shows high yield and good repeatability. The fabricated MEMS energy harvester can operate at ultralow resonant frequencies of 3 and 23 Hz, with an NPD index of 1.73 μW/cm^3^/g^2^ @ 3 Hz. The factors underlying output power degradation in the low-frequency range and potential enhancement strategies are discussed. This work offers new insights into achieving MEMS-scale energy harvesting with ultralow frequency response.

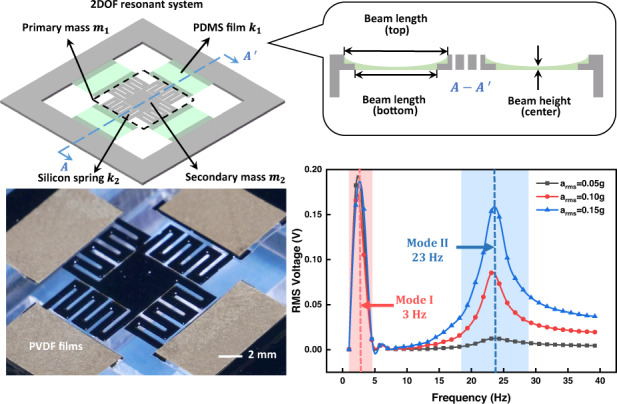

## Introduction

Autonomous systems require microscale energy harvesters, but thus far, microelectromechanical system (MEMS) vibration energy harvesters inadequately satisfy this need. Most MEMS devices are based on resonant structures, which inherently show high resonant frequency (typically hundreds or thousands of Hz) and narrow bandwidth. However, vibrations in the ambient environment are mainly concentrated in the low-frequency (<100 Hz) or even ultralow-frequency (<10 Hz) spectra^1^. This frequency mismatch problem results in a drastic decrease in output power and thus limits the application of MEMS vibration energy harvesters. Therefore, enhancing the ability to resonate in the lower and wider frequency range is a critical issue for MEMS energy harvesters.

The majority of reported MEMS energy harvesters utilize silicon springs with resonant frequencies of hundreds of Hz. For instance, Jia et al. explored and validated an optimal proof-mass-to-cantilever-length ratio for power maximization at a resonant frequency of 210 Hz^2^. Yu et al. proposed a cantilever array with a large silicon-proof mass to enhance the scavenged energy, which resonated at 234.5, 2138.6, and 4057.4 Hz, respectively^3^. Some works have tried to decrease the resonant frequency by changing the structural parameters. Matova et al. investigated whether tapered beams with a small length–width ratio decreased the resonant frequency, but found that they also reduced the output power^4^. Lueke et al. proposed energy harvesters with various shapes of “zigzag” springs. By increasing the equivalent beam length, the resonant frequency of the device decreased to 45 Hz^5^. However, changing the cantilever structural parameters showed a limited effect in reducing the resonant frequency to the ultralow-frequency range and risked inducing structural reliability problems^6–8^.

A few methods have been explored to alleviate the frequency mismatch problem. Three of the most widely used are frequency upconversion^9–12^, bistability^13–18^, and multimodal systems^19–22^. The frequency upconversion method usually consists of two resonators. The lower resonant frequency component responds to external low-frequency excitations and impacts, while the higher resonant frequency component induces high-frequency self-oscillation after impacts. Liu et al. combined two MEMS resonators in one package to realize a frequency upconversion energy harvester and achieved 618 Hz high-frequency self-oscillation at 36 Hz low-frequency excitation^10^. Although frequency upconversion harvested low-frequency kinetic energy via impacts, a larger device volume was required for the two resonators, and high energy loss existed in the impact process. For the bistable mechanism, snap-through actions between two potential wells promised sufficient displacement and velocity. Xu et al. designed and fabricated a buckled beam MEMS energy harvester^13^, which did not rely on structural resonance but rather operated with beam snapping motion when the input energy exceeded a threshold. However, as a nonlinear Duffing resonator, the disadvantage of the bistable device was that it required high-level excitation to overcome the threshold potential barrier. Multimodal systems have at least two subsystems corresponding to two equivalent degrees of freedom (DOFs) and thus can resonate at multiple mode frequencies. Tao et al. proposed a 2DOF MEMS energy harvester with impact-induced nonlinearity, which improved energy harvesting efficiency and achieved multifrequency resonance at two frequencies of 590 and 731 Hz^19^. All the above approaches ameliorated the ability of MEMS energy harvesters to scavenge low-frequency vibrations to certain extents but did not completely resolve the frequency mismatch problem.

The most straightforward and effective solution to the frequency mismatch problem is to reduce the resonant frequency of MEMS energy harvesters to the target level, i.e., from the typically hundreds of Hz level to the tens of Hz level. One of the most promising techniques applies soft and flexible materials with low Young’s modulus to decrease the device resonant frequencies. The loss factor of flexible materials is typically larger, which results in resonators with flexible material presenting a relatively low power output because of the existence of large damping in vibration. However, from another perspective, these resonators present advantages in bandwidth. In addition, they can avoid the structural damage caused by dynamic stress reaching the device limit. To date, only a few energy harvesters based on this design concept have been reported. Yeo et al. proposed a piezoelectric-compliant energy harvester using bimorph PZT films on flexible nickel foils, which achieved low-frequency resonance at 6.3 Hz^23^. Tsukamoto et al. realized a bimorph piezoelectric vibration energy harvester with a flexible 3D meshed-core structure to resonate at 18.7 Hz^24^. Li et al. reported a nickel cantilever based on a polydimethylsiloxane (PDMS) film utilizing frequency upconversion to realize a flexible structure for energy harvesting at 5 Hz^25^. However, these works primarily demonstrate assembled devices, which are still distant from the goal of integrated systems. A critical integration challenge is the incompatible fabrication of soft materials with silicon micromachining processes. Despite efforts such as fabricating resonators using SU-8 photoresist^26,27^, Young’s modulus of SU-8 is still too high to decrease the resonant frequency to the ideal low level for MEMS energy harvesters. Therefore, the compatible fabrication of soft materials on wafers is still a significant challenge. Proper addressal of this issue will offer great potential for integrating ultralow frequency power sources with on-chip power conditioning parts, such as micro energy storage and power management circuits^28–30^.

In this work, a 2DOF MEMS vibration energy harvester, which achieves ultralow-frequency resonance, is proposed and fabricated. Polydimethylsiloxane (PDMS) is used to constitute the suspended beams of the primary subsystem due to its low Young’s modulus of ~750 kPa. The silicon beams, as the elastic part of the secondary subsystem, are designed as zigzag shapes to increase the effective beam length. By cascading the two subsystems, the energy harvester reveals a multimodal response in the frequency spectrum to broaden its working bandwidth. Specifically, a silicon-based compatible fabrication of PDMS is leveraged to realize the MEMS energy harvester structure cascading suspended flexible PDMS and zigzag silicon beams.

## Device design

### Configuration design

In the theory of structural mechanics, the first mode angular resonant frequency *ω*_1_ of a simply supported beam is determined by Eq. (1) (details in Supplementary Information):

1 $$\omega _1 = 2.454\frac{{2\pi }}{{l^2}}\sqrt {\frac{{Ebh^3}}{{12m}}}$$

where *l* is the beam length, *b* is the beam width, *h* is the beam height, *m* is the equivalent proof mass, and *E* is Young’s modulus of the material.

**Fig. 1:**
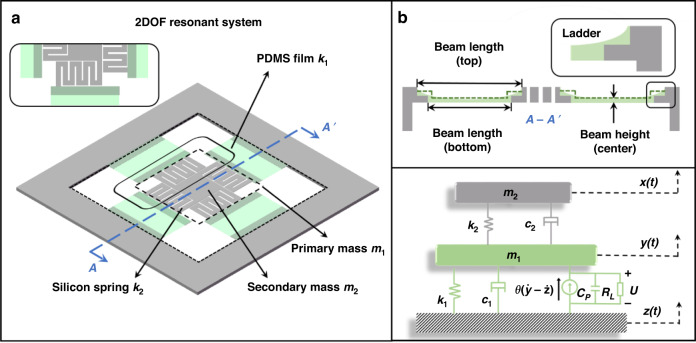
Diagram for the energy harvesting device structure and lumped model. **a** 3D schematic diagram of the 2DOF MEMS vibration energy harvester with corresponding lumped model parameters. **b** Section diagram of the PDMS suspended beam. The dotted line represents the ideal surface of the PDMS suspended film, and the inset is a partially enlarged view of the ladder structure with the actual meniscus PDMS surface. **c** The 2DOF lumped model with the electromechanical coupling of the energy harvester

Equation (1) shows three effective ways to lower the resonant frequency: elongating the beam length, increasing the proof mass weight, and reducing the cantilever’s Young modulus. Utilizing two of the above methods, Fig. 1a depicts the schematic of the proposed MEMS energy harvester. The primary subsystem comprises four thin suspended flexible PDMS beams, in which the low Young’s modulus of PDMS decreases the resonant frequency. The secondary subsystem serves as a proof mass of the primary subsystem. In the secondary subsystem, four silicon beams hang around the central proof mass, and the silicon beams are designed as zigzag shapes to increase the equivalent beam length within limited areas, which also helps to reduce the resonant frequency. The silicon beam at each side can expand to 21 mm due to the zigzag shape (4.8 mm along the PDMS beam width direction and 16.2 mm along the PDMS length direction). Rather than adhering to the silicon supporting layer beneath, this PDMS beam is designed to be an independently suspended beam to construct a truly soft cantilever. As shown in Fig. 1b, to support and connect the suspended PDMS beams, the edges of both silicon frame and silicon zigzag beams are designed as ladder configurations with overlap areas of ~8 × 1 mm^2^. Ideally, PDMS in the overlapped area should exhibit a step profile, as indicated by the dotted line in Fig. 1b. However, in reality, due to the blocking effect by the vertical ladders during the PDMS spin coating process, the surface of the PDMS exhibits a meniscus shape, as shown in the enlarged inset in Fig. 1b. The thickness of the central suspended PDMS beam is only 10 μm. The main structural parameters are listed in Table 1. All four PDMS beams in the device are capable of scavenging energy. The occupied space for a single beam is only 0.03 cm^3^.

**Table 1:** Device structure parameters

Area (mm^2^)		Ladder (mm)		PDMS suspended beams (mm)		Silicon zigzag beams (μm)	
Device	20 × 20	Length	8	Top/Bottom length	5/3	Width	480
Inner frame	10 × 10	Width	1	Width	8	Height	70
Proof mass	4 × 4	Height	0.035	Height (center)	0.01	Gap	320

## Mode frequency analysis

The lumped parameter model of the proposed 2DOF energy harvester is shown in Fig. 1c, with key parameters illustrated in Fig. 1a. When applying piezoelectric layers on the PDMS surface, the complete lumped model of the two-stage system can be expressed as

2 $$m_1\ddot y + c_1(\dot y - \dot z) + k_1(y - z) + c_2(\dot x - \dot y) + k_2(x - y) = {{\theta} {U}}$$

3 $$m_2\ddot x + c_2(\dot x - \dot y) + k_2(x - y) = 0$$

where *m*_1_, *m*_2_, *c*_1_, *c*_2_, *k*_1_, and *k*_2_ are the concentration mass, damping coefficient, and spring stiffness of the primary and secondary subsystems, respectively. *x*, *y*, and *z* represent the displacements of the secondary system, the primary subsystem, and the base excitation, respectively. Parameter *θ* is the electromechanical conversion coefficient, and *U* is the electric potential due to the piezoelectric effect.

The internal equivalent circuit model of piezoelectric energy harvesters usually contains a current source and a capacitor^31,32^. According to Kirchhoff’s law, the electrical output behavior can be represented as:

4 $$C_{\rm {p}}{\dot {U}} + \frac{U}{{R_{\rm {L}}}} + \theta (\dot y - \dot z) = 0$$

where *C*_p_ is the internal capacitor of the energy harvester and *R*_L_ is the external load.

When the parameters above are specified, solving the differential Eqs. (2)–(4) obtains the system’s dynamic response. Further analysis in the frequency domain obtains the system’s spectrum, which has two peaks corresponding to the two resonant modes.

**Fig. 2:**
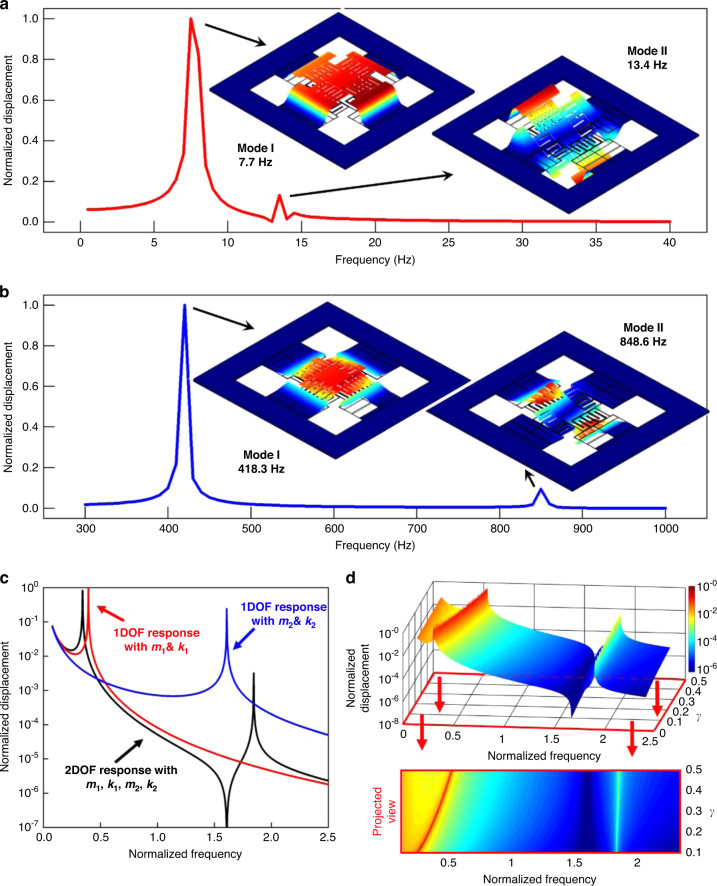
Numerical simulation results via finite element analysis. **a** PDMS for primary subsystems and silicon for the secondary subsystem. (Mode I: 7.7 Hz, Mode II: 13.4 Hz) **b** Silicon for both primary and secondary subsystems. (Mode I: 418.3 Hz, Mode II: 848.6 Hz). **c** Response comparison of two 1DOF systems and the cascaded 2DOF system. The red line represents the 1DOF response with *m*_1_ and *k*_1_ only; the blue line represents the 1DOF response with *m*_2_ and *k*_2_ only; the black line represents the 2DOF response with *m*_1_, *k*_1_, *m*_2_, and *k*_2_. **d** 3D diagram of normalized displacement versus normalized frequency and *γ* to explain the variation trend of modes and its projected view. The resonant frequency of the first mode decreases intensively as the spring stiffness ratio *γ* decreases. In this figure, all the displacements are normalized by the maximum value in the specific subplot

In this work, finite element analysis by COMSOL Multiphysics is adopted to obtain numerical data for the system’s dynamic behaviors. Specifically, the response displacement of *y* in the frequency sweep process is simulated to investigate the eigenfrequency of the system. As shown in Fig. 2a, two peaks exist in the response displacement curve at 7.7 and 13.4 Hz, respectively, corresponding to the first two resonant modes, which are both located in the low-frequency range. At mode I (7.7 Hz), the four PDMS-suspended beams deflect in the same direction. At mode II (13.4 Hz), two opposite PDMS beams deflect up and down, while the other two opposite beams exhibit torsional motions. There is also mode III (14.2 Hz), which is very close to mode II in the frequency spectrum. Although mode III is of limited magnitude, it may expand the bandwidth to some extent. Mode I has a larger response displacement than mode II due to vertical translation rather than rotation movement. The two modes extend the frequency range of the dynamic response, avoiding the case of power generation at only one resonant frequency. In practice, additional modes can be incorporated and optimized to further improve the broadband response. Furthermore, to investigate the low-frequency effect of PDMS-suspended beams, a similar model with an identical structure but different material is also simulated for comparison. As shown in Fig. 2b, the PDMS material of the primary subsystem is replaced by silicon material. Due to the high Young’s modulus of silicon of ~170 GPa, the resonant frequency of the device significantly increased to 418.3 Hz for mode I and 848.6 Hz for mode II, which in turn proves the superiority of the suspended PDMS beams for low-frequency characteristics.

In addition to structural modal analysis, the frequency tuning effect in the lumped model has been further investigated. For two independent 1DOF systems, the relation between mass *m*_1DOF,*i*_, spring stiffness *k*_1DOF,*i*_, and natural angular frequency *ω*_1DOF,*i*_ in a lumped system can be presented as

5 $$\omega _{1{{{\mathrm{DOF}}}},i} = \sqrt {\frac{{k_{1{{{\mathrm{DOF}}}},i}}}{{m_{1{{{\mathrm{DOF}}}},i}}}} ({{{i}}} = 1,2)$$

where *i* = 1,2 marks the parameters for the *i*th independent 1DOF system. Figure 2c compares the simulated responses of the two independent 1DOF systems and the cascaded 2DOF system if *m*_i_ = *m*_1DOF,*i*_ and *k*_i_ = *k*_1DOF,*i*_. The frequencies are normalized by the average resonant frequency of the two 1DOF systems. Compared with the two 1DOF systems, the first mode of the cascaded 2DOF system left shifts to the lower frequency with an intense response, while the second mode right shifts to a higher frequency with a decreased response, proving that the first mode is dominant in the 2DOF system. Even though the gap between the two modes increases, the dominant first mode is of a lower frequency. A more comprehensive numerical simulation is shown in Fig. 2d to investigate the extent of frequency tuning via lumped parameter change, especially by reducing the spring stiffness coefficient of the primary subsystem, such as applying the flexible PDMS beams. Rather than quantifying specific values of the lumped parameters, three major dimensionless ratios in the 2DOF system are defined for the two subsystems: frequency ratio *α* = *f*_1_*/f*_2_, mass ratio *β* = *m*_1_*/m*_2_, and spring stiffness ratio *γ* = *k*_1_*/k*_2_. Here, *f*_1_ and *f*_2_ are the nominal resonant frequencies of both subsystems assumed to be independent 1DOF systems. Based on Eq. (5), the relation between the defined three ratios can be expressed as

6 $$\alpha ^2 = \frac{\gamma }{\beta }$$

Since the mass of the primary subsystem includes the secondary subsystem (*β* > 1) and the material for the primary subsystem has a lower Young’s modulus (*γ* < 1), *α* is always <1. Keeping *m*_1_, *m*_2_, and *k*_2_ at constant values and adjusting only *k*_1_, the normalized displacement is simulated with *γ* ranging from 0.1 to 0.5, and the frequencies are still normalized by the average resonant frequency of the two peaks. The 3D visualization results and its corresponding projected view are both presented in Fig. 2d. Different from a slight tuning effect on the 2nd resonant frequency, the effect of reducing the dominant 1st resonant frequency by a smaller *γ* is significant. These results validate the contribution of low Young’s modulus materials such as PDMS to the reduction of the dominant resonant frequency of the multimodal device.

## Fabrication

**Fig. 3:**
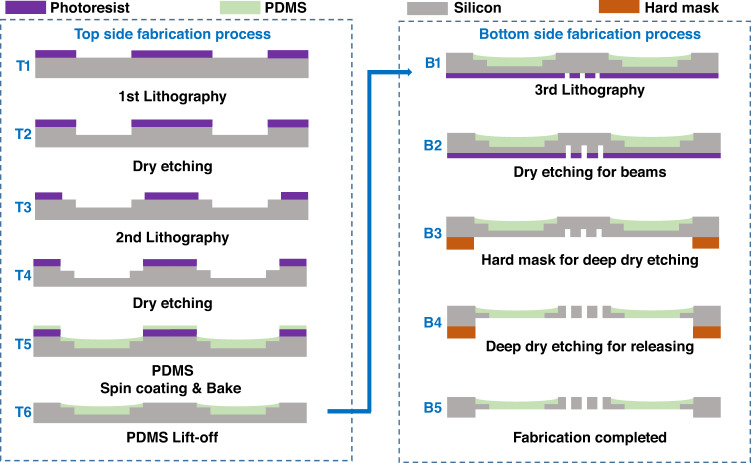
Fabrication process flow of the proposed two-stage MEMS energy harvester device. T1–T6: Top-side fabrication process primarily for PDMS-suspended beams. B1–B5: Bottom side fabrication process primarily for silicon zigzag beams

The MEMS energy harvesters are fabricated using a microfabrication process on 300 μm thick, 4 inch, <100> silicon wafers. The detailed fabrication process flow, which contains the top side and bottom side processes, is presented in Fig. 3. Various fabrication methods for PDMS have been reported in the last decade^33–35^, but few have realized patterning and release of suspended PDMS thin films. Considering the difficulty of simultaneous compatible fabrication of PDMS-suspended beams and silicon zigzag beams, the two subsystems patterning process is intentionally designed on the top and bottom sides, respectively.

The top-side process steps T1–T6 primarily describe the fabrication of PDMS suspended beams, which includes ladder structure etching, PDMS spin coating, baking, and lift-off. The T1–T4 steps, where lithography and dry etching are performed twice, respectively, fabricate ladders to guarantee a sufficient overlap area between the silicon frame and PDMS beams so that the flexible beams hang in the air. The dry etching process is realized by SF_6_ gas, and the etching is anisotropic (with a degree of anisotropy 0.7–0.8) with a relatively fast rate of ~1.2 µm/min. The PDMS mixture is prepared in advance before step T5. Then, this PDMS mixture is spin-coated and baked to be cured in step T5. Afterward, PDMS patterning is completed after the lift-off process in step T6.

Patterning PDMS thin film is the precondition for the compatible fabrication of PDMS and silicon beams. Different from traditional fabrication methods such as casting molding or dry etch patterning, a novel PDMS lift-off process is proposed, which realizes confined shapes of PDMS thin films on wafers with high fidelity. To solve the difficulty of PDMS adhesion at the pattern edge, the proposed process addresses the following two key points: (1) Reducing the viscosity of PDMS by dilution. In addition to the traditional mixture of PDMS base and curing agent at a 10:1 weight ratio, tert-butyl alcohol (TBA) is used as a solvent (1:4 weight ratio with PDMS mixture) to reduce the viscosity of PDMS. Then, the mixture is placed in a vacuum chamber for half an hour to remove the air bubbles caused by mixing. Afterward, diluted PDMS with no air bubbles is spin-coated onto a layer of patterned photoresist and baked at 80 °C until it is completely cured. Then, the wafer is immersed in acetone to realize the lift-off process. (2) Increasing the step height difference at the pattern edge to force rupture of the PDMS film. In the traditional lift-off process, the step height is realized by photoresist thickness in the range of 1–20 μm. However, for a thick PDMS film (10 μm) with high viscosity, the photoresist thickness alone is not enough to force the PDMS film to rupture at the pattern edge and results in lift-off failure. Therefore, in this work, we increase the step height by increasing photoresist thickness with the etching depth. As shown in T4, an additional 35 μm silicon is etched at the pattern edge. Together with the 15 μm photoresist thickness, a total step height difference of 50 μm is achieved, which is large enough to fulfill effective lift-off of the PDMS thin films. (More details are provided in the Supplementary Information.)

To avoid direct lithography on cured PDMS, the silicon zigzag beam fabrication process comprising steps B1–B5 is carried out on the bottom side. With double-side lithography, the silicon zigzag beam is patterned on the bottom side as in Step B1. The etching of the silicon beams can be divided into two procedures: (1) Pre-etching, in which 80 μm silicon is dry etched to form the zigzag shapes in Step B2. Here, an 80 μm etching depth is adopted to be slightly larger than the silicon beam height of 70 μm and guarantee complete release in the next step. (2) Hard mask etching, in which the stainless steel hard mask protects the silicon frame while 230 μm silicon is dry etched for ~3.5 h until the PDMS beams are revealed, as illustrated in steps B3 and B4. The silicon zigzag beams are simultaneously released in this process. As a consequence, the PDMS beams are suspended in the air with support from the ladders on the silicon frame and silicon zigzag beams, as shown in step B5.

**Fig. 4:**
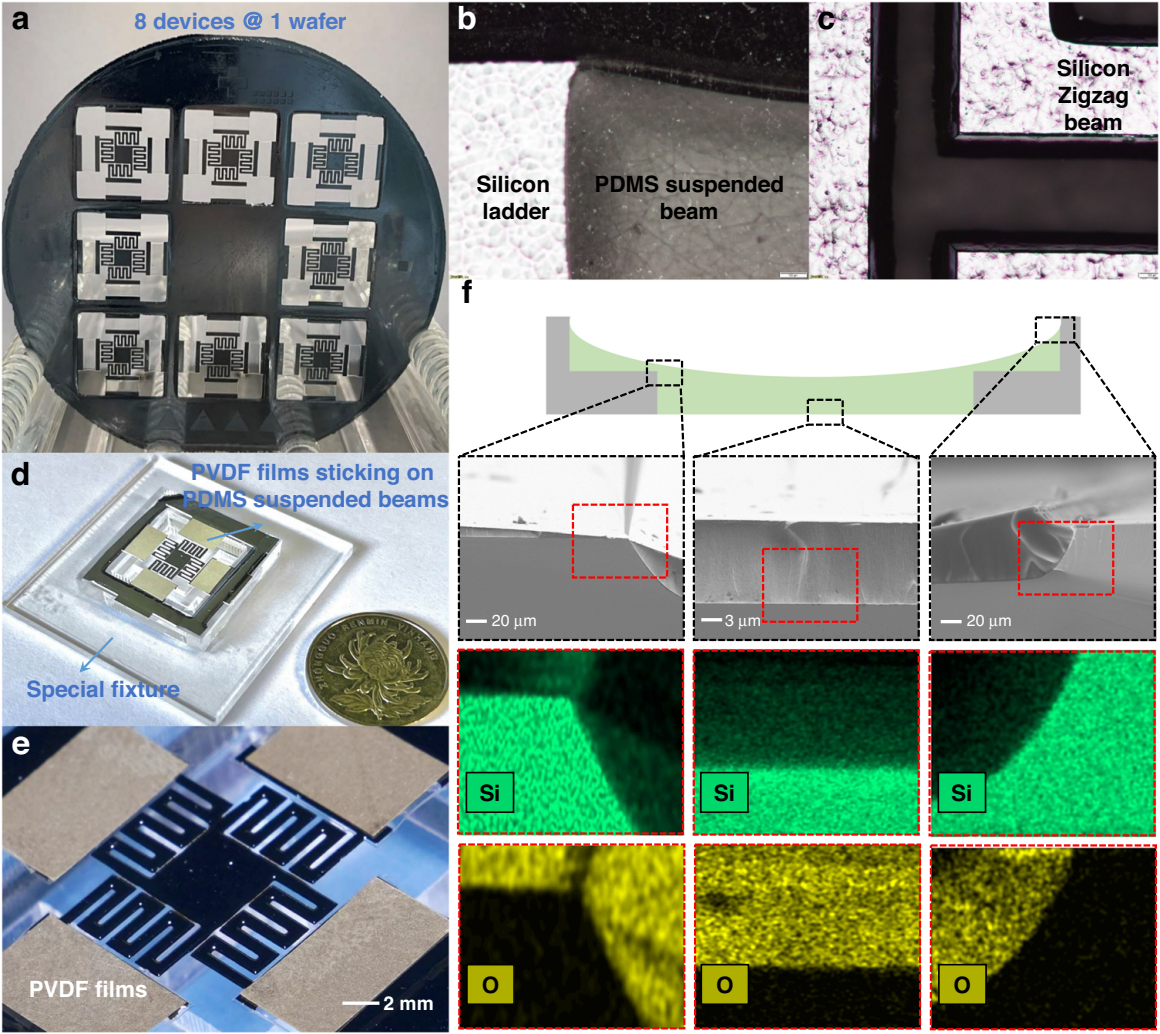
Characterization for the energy harvesting device and its core parts. **a** Fabricated on-chip energy harvester devices. **b** Microscope detailed picture of PDMS suspended beams. **c** Microscope detailed picture of silicon zigzag beams. **d** Photograph of the whole energy harvester device on a specially customized fixture with attached piezoelectric PVDF films in detail. **e** Photograph of the core part of the energy harvester device in detail. **f** SEM section images and elemental analysis (Silicon and Oxygen) of PDMS on the first ladder, second ladder, and center of suspended beams

Figure 4 presents the fabricated 2DOF energy harvester with cascaded beams and the details of its morphology and component characterization. Figure 4a shows eight devices fabricated on one 4-inch silicon wafer. Figure 4b and c are the microscope photographs of the overlap area of PDMS, adjacent silicon ladders, and the local region of zigzag silicon beams, respectively. In the micrographs, it could be observed that the PDMS film homogeneously covers the overlap area. Figure 4d presents the details of the integral device after applying PVDF piezoelectric thin films over the suspended PDMS beams. Figure 4e shows the core part of the energy harvester device in detail. Figure 4f shows the SEM images as well as the element distribution of PDMS at three critical positions: the upper ladder, the lower ladder, and the center of the suspended beams. The characterization confirms the successful lift-off of large-area PDMS films on the wafer and the effective connection between the PDMS-suspended film and silicon ladders.

## Characterization and discussion

### Experimental results

**Fig. 5:**
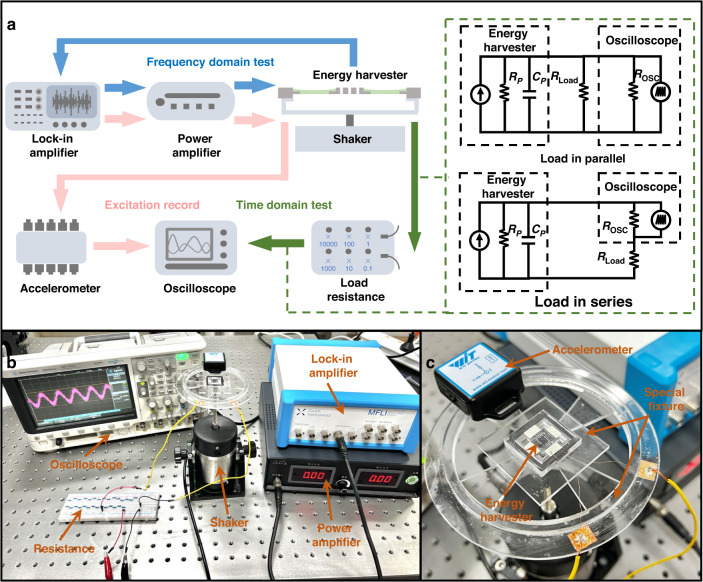
Setup for experimental tests. **a** Schematic diagram of the experimental setup and connection relationship. The blue arrow represents the frequency domain test, the green arrow represents the time domain test, and the pink arrow represents the excitation measurement and recording. The illustration shows two test connection methods between the energy harvester, oscilloscope and load resistance: load in parallel with the oscilloscope and load in series with the oscilloscope. **b** Photography of the actual experimental setup including all test instruments in detail. **c** Photography of the energy harvester and accelerometer fixed on a special fixture for tests

The fabricated energy harvester is attached to a special fixture and then adhered to a shaker. The equipment setup, connections and testing methods are shown in Fig. 5a. The circuit diagrams illustrated in Fig. 5a depict the serial and parallel connections of the oscilloscope (1 MΩ input resistance) with the load resistance. These two testing circuits guarantee a sufficiently large variation range of the total resistance of the energy harvester and avoid inaccurate measurement caused by the possible excessive load resistance. Figure 5b, c show photos of the actual experimental setup in detail. A sinusoidal excitation signal is generated by the lock-in amplifier (Zurich Instruments MF-DEV5908) and amplified by the power amplifier (Beijing HYZX GF-100) to drive the shaker (Beijing HYZX JZ-2). Commercial flexible PVDF piezoelectric films (PolyK 1-1004347-0) are stuck on the surface of the PDMS-suspended beam to scavenge vibration energy. The commercial PVDF piezoelectric film has top and bottom Ag electrodes and an overall thickness of 28 μm. This thickness somewhat changes the stiffness of the primary subsystem, which may be a cause of the deviation between the simulated and experimental results. An accelerometer (WitMotion WT901BLE5.0C) measures the excitation of the energy harvester. Frequency domain and time domain data are acquired by the lock-in amplifier and oscilloscope (Agilent Technologies DSO-X 2014A), respectively. Considering that the structure is symmetrical, only one beam is tested for characterization, and the performance of the entire device can be estimated as approximately fourfold that of the single beam.

**Fig. 6:**
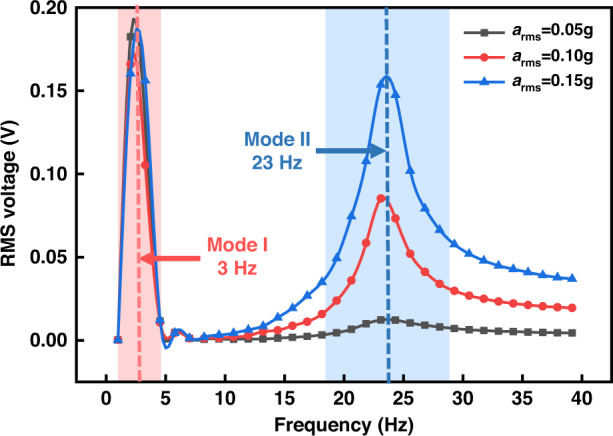
Frequency response of the energy harvesting device. Measured voltage outputs in frequency sweep from 1 to 40 Hz under excitations of 0.05, 0.10, and 0.15 g by the lock-in amplifier. The two peaks correspond to two resonant modes. (Mode I for 3 Hz and Mode II for 23 Hz)

The frequency-domain test results are shown in Fig. 6. The frequency sweeping ranges from 1 to 40 Hz under excitations of 0.05, 0.10, and 0.15 g, respectively, which represent extremely low vibration levels. The measured curves exhibit two distinct peaks, demonstrating the corresponding two resonant modes. Mode I occurs at 3 Hz, while mode II occurs at 23 Hz. Notably, the intensity of the output voltage under mode I is stronger than that under mode II due to a large displacement response under vertical translation, which is consistent with the simulation. As the excitation acceleration increases, the response of mode I slightly decrease, while that of mode II increases, showing that these two modes are dynamically correlated. A complete investigation of the interactions between the two modes requires a further increase in excitation acceleration; however, limited by the shaker motion displacement, the maximum attainable acceleration in this work is 0.15 g in the 1–40 Hz frequency range. Another interesting observation is the wider bandwidth at mode II, which possesses several possible explanations: (1) The wider bandwidth at 23 Hz may be induced by the flexibility of PDMS material accompanied by high losses, which suggests high damping in mechanical vibrations and low-quality factor *Q*. A resonator composed of flexible materials with low-*Q* factors may exhibit relatively low output but would offer advantages in bandwidth. (2) The expansion of bandwidth may also derive from the diversified motions of the PDMS beams in mode II. From the vibration mode diagram in Fig. 2a, at 3 Hz, the four PDMS beams deflect in the same bending direction, which explains the linear response resulting in a narrow bandwidth. However, at 23 Hz, a pair of opposite beams exhibit reverse bending motions, while the other pair of opposite beams exhibit torsional motions because of the flexibility of PDMS. (3) The existence of mode III, in which the device vibration mode deformation is consistent with that of mode II (opposite in pairs) and the resonant frequency is very close to that of mode II in the frequency spectrum, may also contribute to the wider bandwidth. Deviations from the simulated resonant frequencies exist, and the potential causes are variations in the structure, material, and process. On the one hand, the true structure of the suspended PDMS beam appears as a meniscus, yet is represented by a regular film in simulation. On the other hand, the elastic characteristic of PDMS may change due to the dilution effect of TBA. Nevertheless, its experimental frequency response demonstrates that the device works effectively in the ultralow frequency range.

**Fig. 7:**
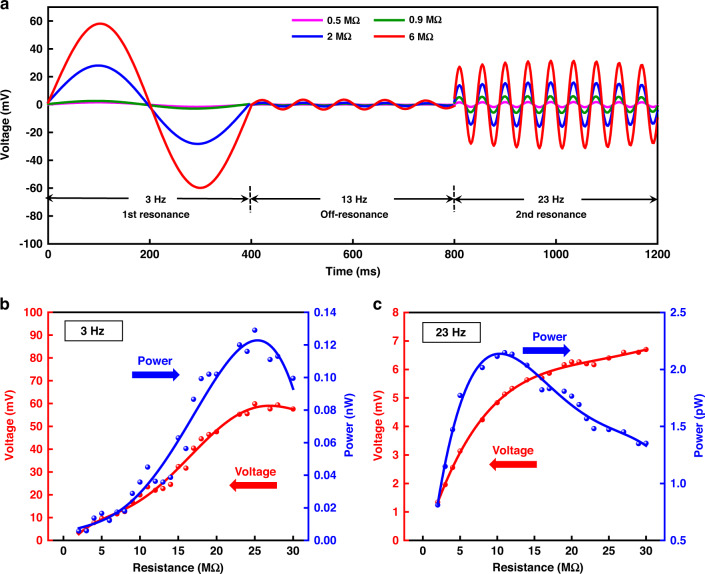
Time-domain tests and load resistance optimization tests. **a** Measured time-domain output voltage response under 0.1 g excitation with different load resistances at 3, 13, and 23 Hz. **b** Measured voltage and power outputs under 0.05 g excitation with different load resistances at the 1st resonant frequency of 3 Hz. **c** Measured voltage and power outputs under 0.05 g excitation with different load resistances at the 2nd resonant frequency of 23 Hz

The measured time-domain output voltage response under 0.1 g excitation with different load resistances is presented in Fig. 7a. The internal resistance of the oscilloscope is 1 MΩ, which is insufficiently large with respect to the output impedance of the piezoelectric element. To guarantee a comprehensive characterization for a broad range of load resistance changes, two testing connections in Fig. 5a are adopted. The curves are tested under load resistances of 1 and 10 MΩ in parallel and 1 and 5 MΩ in series. The equivalent resistances are 0.5, 0.9, 2, and 6 MΩ, respectively. The two resonant frequencies, 3 and 23 Hz, corresponding to the two peaks in Fig. 6 are set as the frequency of the vibration excitation. In addition, the frequency of 13 Hz is investigated as a contrasting configuration, which is supposed to demonstrate a weaker response due to its off-resonance characteristic. Figure 7a compares the time domain response under the three frequencies. The results prove that the responses under 3 and 23 Hz are significantly stronger than that under 13 Hz, and the response of mode I is more intense than that of mode II. The result is essentially consistent with the simulation and frequency domain test results.

The impedance characteristics at 3 Hz (mode I) and 23 Hz (mode II) are also investigated. The test vibration excitation remains at a root mean squared value of 0.05 g. As shown in Fig. 7b, a maximum output voltage of 60 mV and a maximum output power of 0.13 nW (~0.52 nW for four beams in the device) are obtained with an optimum load resistance of ~25 MΩ at the first mode resonant frequency of 3 Hz. In Fig. 7c, a maximum output voltage of 7 mV and a maximum output power of 2.3 pW (9.2 pW for four beams in the device) are obtained with an optimum load resistance of ~12 MΩ at the second mode resonant frequency of 23 Hz.

We note that the output voltage of this device is comparable to that of most MEMS vibration energy harvesters. The relatively low output power is partly due to the extremely low excitation level, which is restricted by the shaker motion limit at the low-frequency excitation (details in Supplementary Information). Another major cause of the limited output power is that a lower resonant frequency leads to a larger internal resistance. At the low-frequency range, the influence of the device’s internal resistance on output power is worth discussing. When the resonant frequency is in the hundreds and thousands of Hz, the internal capacitive reactance, 1/*jωC*, of the equivalent circuit model of the energy harvester is quite small (typically kΩ level) due to the high frequency. However, when the resonant frequency reaches the ultralow level (<10 Hz), the internal capacitive reactance is too large to be ignored, resulting in a rapid increase in both the internal and optimal loads to the MΩ level. Usually, to alleviate the influence of capacitance, reactance cancellation is a useful method. However, it is minimally applicable in the ultralow frequency range because it requires impractically high inductance. In this work, the optimal load resistance rises from 12 to 25 MΩ when the resonant frequency decreases from 23 to 3 Hz, proving that the internal resistance increases in the ultralow frequency range. Since the maximum power is inversely proportional to the optimal load, under comparable output voltage, the maximum power is decreased by almost three orders of magnitude when the optimal resistance increases from the kΩ level to the MΩ level. Therefore, to solve the mismatch frequency problem with environmental vibration, concurrent efforts are required to decrease the device resonant frequency as well as to increase the internal capacitive reactance. These advancements will maintain the optimal resistance in a relatively low range and can thus enhance the output power to a practical level while remaining in the ultralow frequency range.

### Performance comparison and outlook

Evaluating the performance of an energy harvester is not straightforward since the resonant frequency, working bandwidth and excitation vibration of various harvesters are distinct. Thus, maximum power and open-circuit voltage are widely used metrics. However, to eliminate the influence of device size and test excitation, normalized power density (NPD) has become a popular evaluating indicator for MEMS energy harvesters, and is presented as follows^36^:

7 $${\rm{NPD}} = \frac{{{\rm{Power}}}}{\mathrm{{Volume}} \cdot {{{\mathrm {Acceleration}}}^2}}$$

The proposed device achieved an NPD of 1.73 μW/cm^3^/g^2^ @ 3 Hz. Table 2 provides a detailed performance comparison with other reported MEMS energy harvesters. Generally, most reported MEMS energy harvesters have resonant frequencies above 30 Hz, and the proposed energy harvester shows a very prominent advantage of operating in the ultralow frequency range. Overall, it can be seen that applying soft material with a compatible fabrication process contributes to the ultralow frequency response, which drives MEMS energy harvesters forward for applications in real environments. However, the output power of this energy harvester is still relatively low. We attribute this to several aspects: (1) The excitation acceleration is restricted by the shaker motion amplitude limit at the ultralow frequency range; (2) The rapidly increasing internal capacitance of the piezoelectric layer in the ultralow frequency range; (3) The piezoelectric polymer PVDF, as an energy transducer, has a limited electromechanical conversion capacity. Therefore, to realize MEMS energy harvesting applications in real environments, further research is needed, especially exploring soft materials with high piezoelectricity and integration capability. It was also challenging, yet quite promising, to develop a compatible microfabrication process incorporating high-performance piezoelectric soft materials into the device structure. This process can potentially boost the output power in the ultralow frequency range to a practical level.

**Table 2:** Comparisons of this MEMS energy harvester with various references

Ref.	Prin.	Vol. (cm^3^)^a^	Acc. (g)	Power (μW)	Voltage (V)	Resist. (MΩ)	NPD (μW/cm^3^/g^2^)	Freq. (Hz)^b^
^20^	EM	0.12	1	128	0.16	0.000038	1064.17	185
^37^	EM	0.13	1.52	0.61	0.018	0.000033	2.03	55
^38^	EM	0.35	1	0.06	0.0038	0.000626	0.16	840
^19^	ES	0.296	1.3	14.8	17.5	5	29.59	736
^39^	ES	0.484	2	20.7	50	50	10.69	110
^40^	ES	0.305	0.5	1.5	7.75	40	19.67	28
^41^	ES	0.14	0.43	0.95	9	30	36.70	95
^42^	ES	0.8	1	54	100	50.5	67.50	139
^3^	PE	0.72	0.5	66.8	7	0.22	370.83	234.5
^12^	PE	0.0087	0.06	0.00117	0.042	1.6	35.12	27.4
^26^	PE	0.0189	4	1.37	1.42	0.36	4.53	580
^43^	PE	0.016	1.0	0.09	0.116	0.33	5.41	36
^44^	PE	0.032	0.25	0.023	0.043	0.04	11.50	68
^45^	PE	0.489	3	321	35	0.14	72.94	100
^46^	PE	1.276	1.5	7.18	2.68	0.5	2.50	406
^47^	PE	0.048	0.2	0.14	0.6	2	70.83	71.8
This work	PE	0.12	0.05	0.00052	0.06	25	1.73	3

*EM* electromagnetic, *ES* electrostatic, *PZ* piezoelectric

^a^Some energy harvester volumes are estimated by area and thickness

^b^The frequency corresponds to the vibration mode with the maximum output if there are multiple modes

## Conclusion

In this work, a 2DOF multimodal MEMS vibration energy harvester cascading flexible PDMS suspended beams and silicon zigzag beams were designed, fabricated, and tested. This device effectively worked in the ultralow frequency range. Finite-element analysis of the device proves that decreasing the spring stiffness of the primary subsystem, which is guaranteed by the low Young’s modulus of soft PDMS material, is a key factor for reducing the device’s first mode resonant frequency. A novel PDMS lift-off process was demonstrated to realize suspended PDMS thin films with confined patterns on wafers, offering compatibility with most micromachining processes. The fabricated MEMS energy harvester exhibited two mode frequencies at 3 and 23 Hz, demonstrating dynamic responses in the ultralow frequency range. The impedance characterization test showed that the device with four beams generates 60 mV, 0.52 nW @ 3 Hz and 7 mV, 9.2 pW @ 23 Hz, corresponding to an NPD of 1.73 μW/cm^3^/g^2^ @ 3 Hz. This work provides new insights for achieving MEMS-scale energy harvesting with ultralow frequency response, explores potential power-limiting problems, and describes prospects for future work to further enhance the output power of MEMS energy harvesters in the ultralow frequency range.

## Supplementary information

Supplementary material
