# Supplementary material for: Multimodal MEMS vibration energy harvester with cascaded flexible and silicon beams for ultralow frequency response
Source: Microsyst Nanoeng. 2023 Mar 23;9:33. doi: 10.1038/s41378-023-00500-8 (PMC10033895; doi:10.1038/s41378-023-00500-8)
Supplement: Supplementary file 1 — Supplementary material [file 41378_2023_500_MOESM1_ESM.docx]

**Supplementary Information**

**Multimodal MEMS vibration energy harvester with cascaded flexible and silicon beams for ultra-low frequency response**

**Haizhao Feng^1^, Ling Bu^2^, Zhangshanhao Li^1^, Sixing Xu^3^, Bingmeng Hu^1^, Minghao Xu^1^, Siyao Jiang^1^ and Xiaohong Wang^1*^**

^1^School of Integrated Circuit, Tsinghua University, Beijing 100084, China.

^2^School of Information Engineering, China University of Geosciences, Beijing 100083, China.

^3^College of Semiconductors (College of Integrated circuits), Hunan University, Changsha, 430001, China.

These authors contributed equally: Haizhao Feng, Ling Bu.

Email: wxh-ime@tsinghua.edu.cn (Xiaohong Wang)

**The natural frequency of simply supported beams**

For a resonator with a complicated structure, it may be hard to get accurate equations to describe the relationship between natural frequency and structure parameters. However, the analysis of the most common simply supported beam structure could serve as guidance. Therefore, the qualitative influence of structural parameters on the natural frequency for the resonator with a complicated shape could be comprehended.

The deflection equation of a simply supported beam is presented below:

$EI\frac{\partial w^{4}}{\partial x^{4}}+m\frac{\partial w^{2}}{\partial t^{2}}=0$ (S1)

where *E* is Young's modulus of the material, I is the section moment of inertia, *w* is the deflection of the beam along the length direction *x*, and *m* is the linear density of the beam.

In the method of separating variables, suppose that:

$W(t,x)=\varphi(x)N(t)$ (S2)

By applying equation (S2), equation (S1) is represented as:

$EI\frac{{\varphi(x)}^{''''}}{\varphi(x)}+m\frac{{N(t)}^{''}}{N(t)}=0$ (S3)

Further suppose that:

$\lambda^{4}=\frac{{\varphi(x)}^{''''}}{\varphi(x)}=-\frac{{mN(t)}^{''}}{EIN(t)}$ (S4)

Equation (S4) can be rearranged as two differential equations, expressed as follows:

${\varphi(x)}^{''''}-\lambda^{4}\varphi\left( x \right)=0$ (S5)

$mN(t) ''+\lambda^{4}EIN(t)=0$ (S6)

The natural frequency of a spring oscillator is expressed:

$f=\frac{1}{2\pi}\sqrt{\frac{k}{m}}$ (S7)

However, equation (S6) corresponds to the form of an undamped vibration dynamic equation, while *λ*^4^*EI* is the elastic coefficient of the system. According to equations (S6) and (S7), the natural frequency of a simply supported beam can be expressed as follow:

$f=\frac{\lambda^{2}}{2\pi}\sqrt{\frac{EI}{m}}$ (S8)

By solving the fourth-order differential equation (S5), the solution is expressed as follows:

$$\varphi\left( x \right)=A_{1}e^{i\lambda x}+A_{2}e^{-i\lambda x}+A_{3}e^{i\lambda x}+A_{4}e^{-i\lambda x}$$

$=B_{1}\sin\lambda x+B_{2}\cos\lambda x+B_{3}\sinh\lambda x+B_{4}\cosh\lambda x$ (S9)

Meanwhile, the boundary conditions of the simply supported beams are presented as follows:

$\left\{ \begin{aligned} \varphi(0)=0 \\ \varphi(0)'=0 \\ \varphi(l)=0 \\ \varphi\left( l \right)^{'}=0 \end{aligned} \right.$ (S10)

According to equations (S9) and (S10), the solution is as follows:

$\lambda_{i}=\frac{\left( i+0.25 \right)\pi}{l} (i=1,2,3\ldots\ldots)$ (S11)

As a result, the natural frequency of a simply supported beam can be represented as follows:

$f=\frac{{(i+0.25)}^{2}\pi}{2l^{2}}\sqrt{\frac{Ebh^{3}}{12m}}$ $(i=1,2,3\ldots\ldots)$ (S12)

where *E* is Young's modulus of the material, *l*, *b*, and *h* are the beam length, width, and thickness respectively. *m* is the linear density of the beam. To decrease the resonance frequency, lower Young’s modulus of the structure material and longer equivalent length both make a contribution.

**Structural parameters optimaztion by simulations**

Typically, frequency tuning by structural parameters optimazation is a common and useful method. Herein, some simulations had been made from two aspects: (1) Common structural optimazition by tuning the length of PDMS and zigzag silicon. (2) Adopting more balanced zigzag structure to avoid rotational movement.

It is really very useful method to adjust the beam length to realize frequency tuning. However, different from the traditional rectangular cantilever beam, the structural parameters are usually interactive rather than independent in a relatively complicated structure. For instance, in a device with a fixed overall area, a longer PDMS cantilever will sharply compress the area of the intermediate secondary subsystem, which means that the length of the zigzag silicon beam will also be reduced, and its lumped mass will also be reduced. Sometimes, it is hard to distinguish which parameter optimization plays the dominant role. To exclude these mutual influencing factors, by control variate method, parameters change independently in the primary subsystem and secondary subsystem, as shown in Fig.S1 & S2. The simulation results shows that the parameter changing in the second subsystem has a very limited influence on resonant frequency performance while the parameter changing in the primary subsystem is decisive due to the very outstanding low-frequency characteristic of PDMS flexible beams. The result is consistent with the conclusion drawn from the simulation of lumped model.


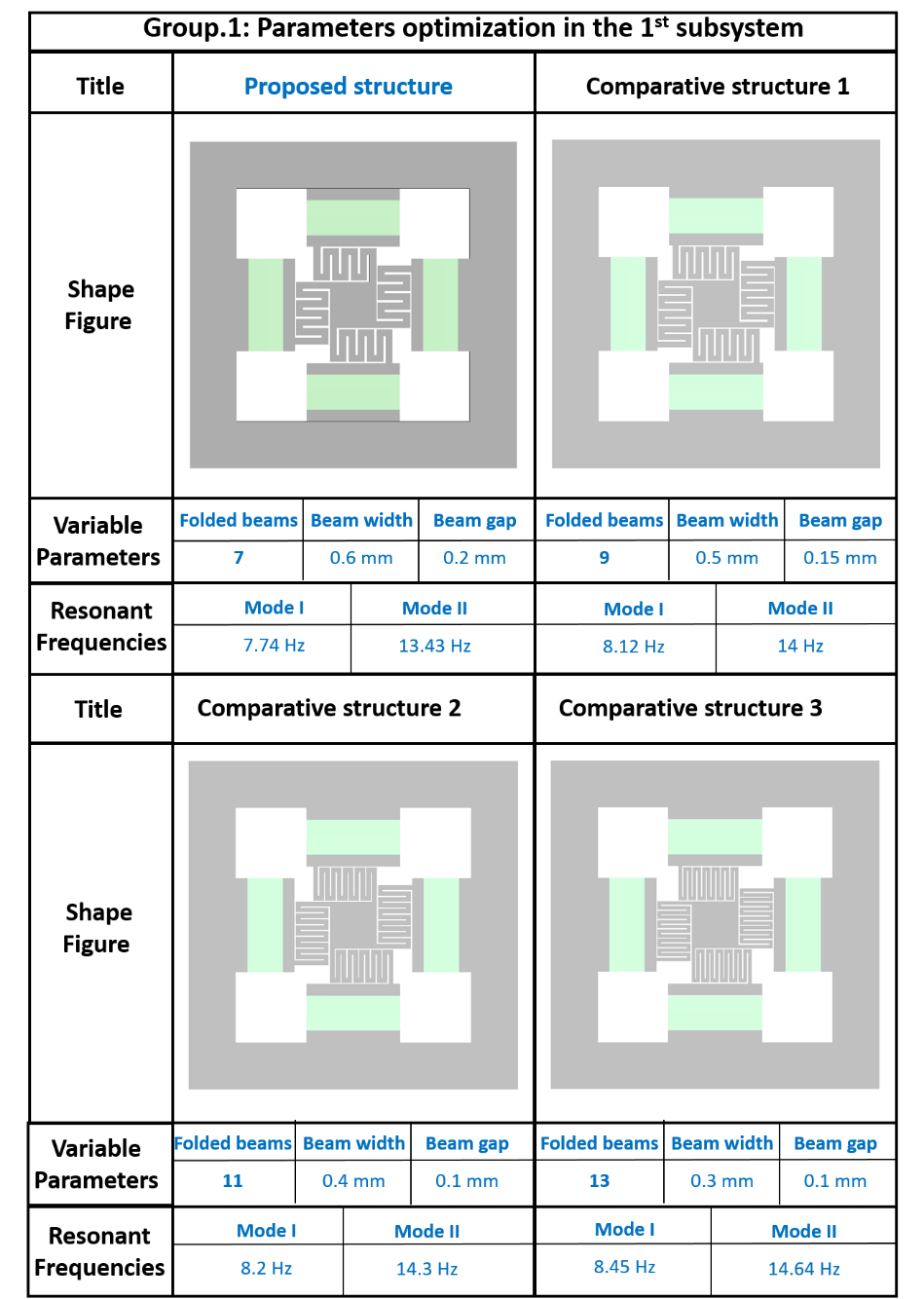


**Fig.S1 Comparison of resonant frequencies performance by parameters optimization in the 1st subsystem.**

**
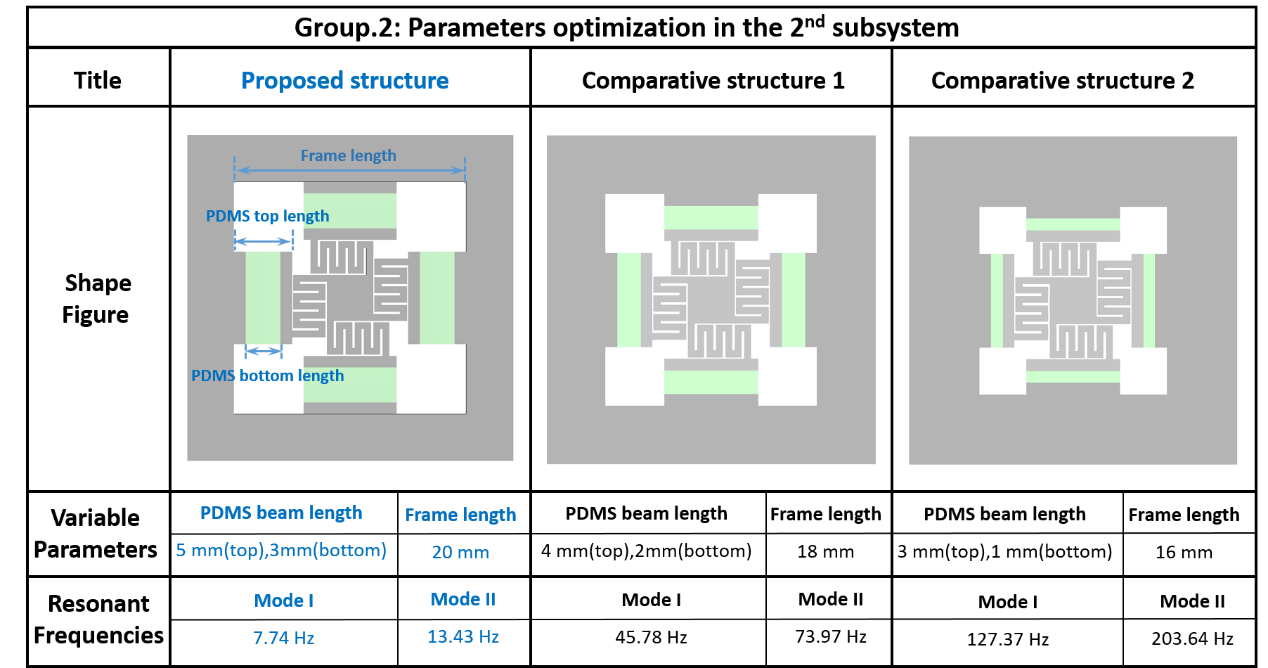
**

**Fig.S2 Comparison of resonant frequencies performance by parameters optimization in the 2nd subsystem.**

Reducing the torsion movement and increasing the bending movement of the beam is a very effective way to improve the output performance by designing more balanced zigzag beams. There is an interesting point worth discussing that the place where piezoelectric materials are applied usually determines the output performance of the device. In other words, in this device, the deformation at the PDMS beam is the main factor that determines the output of the device, seen in Fig.2a. For more balanced zigzag beams to avoid the rotation movement, we took a reported structure for simulations^1^. The results are shown in Fig. S3. From the deformation pictures at mode II, the improved zigzag beams are indeed more balanced so that the four PDMS beams have a synchronous deformation while the PDMS beams present two states (2 bending & 2 rotation) in the proposed structure. However, despite the difference of the axis, the rotation movement occurs for all the structures. That means the rotation of the whole device is mainly induced by the super flexibility of the PDMS beams. The change of the vibration mode of the zigzag silicon beam (torsion or bending) affects the displacement of the seismic mass, affecting the offset of the second resonant frequency point in the spectrum. And the influence on the output may be limited, which is generally contributed by the first resonant frequency point.

**
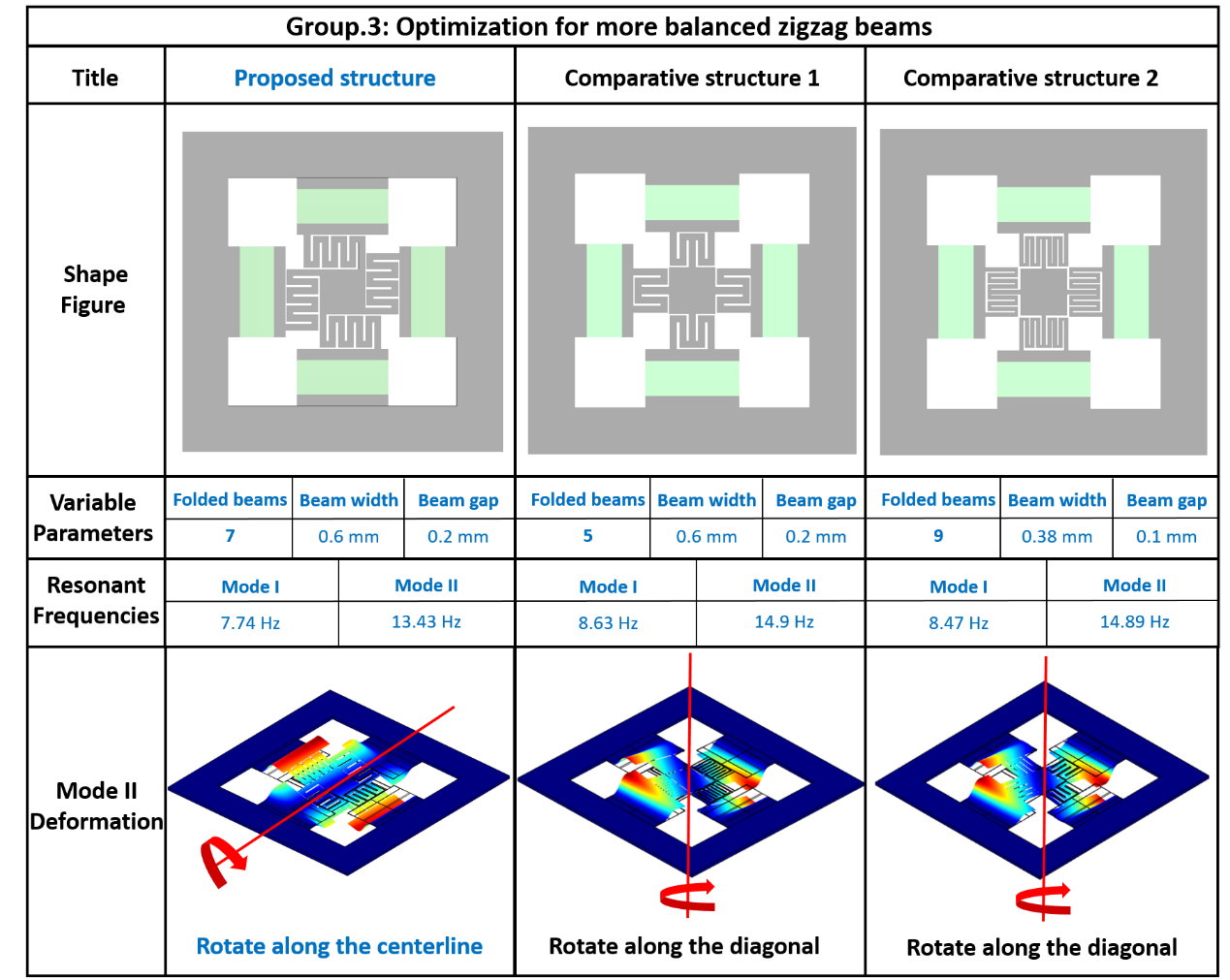
**

**Fig. S3 Comparison for optimization for more balanced zigzag beams.**

**Details about the dry etching process**

The dry etching process is realized by SF6 gas and the etching is anisotropic on the whole (the degree of anisotropy is about 0.7-0.8) with a relatively fast rate of about ~1.2 µm/min（~35 µm/30 min）. The specific etching parameters are shown in the following Table S1.

**Table S1 Dry etching fabrication process parameters**

| Parameters | Stable | RF_On | Etch |
| --- | --- | --- | --- |
| Time (s) | 10 | 10 | 1800 |
| Pressure (mTorr) | 50 | 50 | 50 |
| Gas_SF6 (500SCCM) | 200 | 200 | 200 |
| RF Power (W) | 0 | 300 | 300 |
| Bias RF Power (W) | 0 | 0 | 30 |

**PDMS large-area lift-off fabrication process**

PDMS, as a widely used material, has been applied to many research fields. Although many fabrication approaches have been reported, reliable and easy-operating patterning PDMS film fabrication is still challenging work. Park et al. proposed a PDMS lift-off technique, which can form pattern arrays on PDMS film^2^. In this work, some arrays with small areas were successfully lifted off. However, removing the large area pattern and keeping the small area pattern on the wafer is more difficult work. One of the most critical factors to achieve a large-area lift-off process is to ensure a large enough vertical distance between the patterned layer and the removed layer, which is usually dependent on photoresist thickness. Unfortunately, continuously increasing the thickness of the photoresist is infeasible. Based on this mentality, we explore an on-chip large-area lift-off fabrication technique. By taking advantage of etching depth and photoresist thickness simultaneously, large-area PDMS on photoresist could be peeled off more easily. Fig. S1 shows wafer photographs of each step of the PDMS large-area lift-off fabrication process. From the T5 step in Fig. S1, it is clear to observe an ideal pattern array formed on the wafer as expected.

**
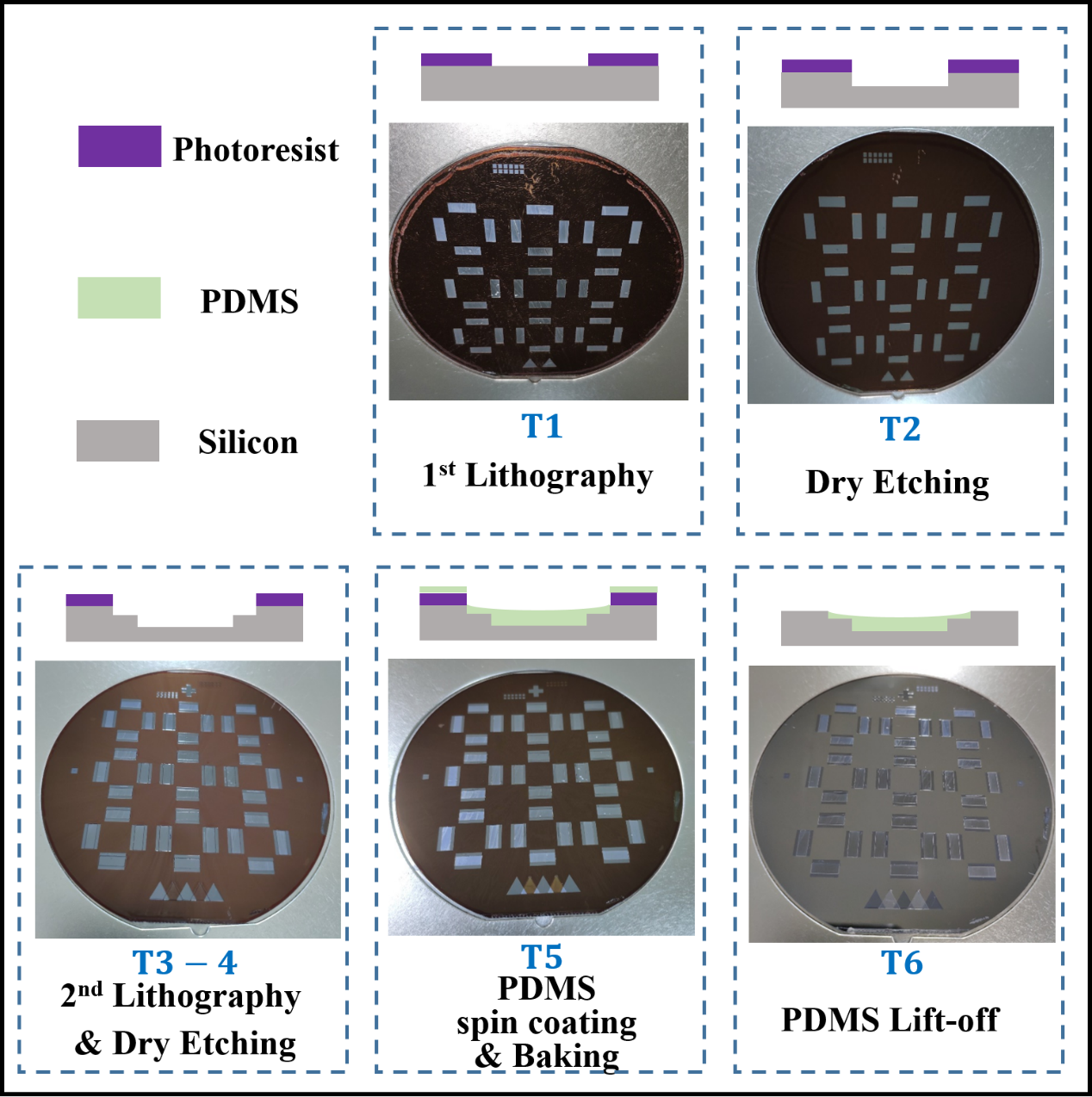
**

**Fig. S4 Wafer photographs of each step of the PDMS large-area lift-off fabrication process.**

**Acceleration tests of the shaker excitation**

Few MEMS energy harvesters have a Hertz-level resonance response. 3 Hz and 23 Hz are resonant frequencies of mode I and mode II, respectively. And the frequency of 13 Hz is selected as a contrast. Tests at the three frequencies above are all under excitations with the same root mean square value, which means that the vibration at a lower frequency requires a larger displacement. Acceleration tests at the three frequencies under excitation of 0.05 g, 0.075 g, and 0.1 g are shown in Fig. S2. Differing from normal test results of frequencies at 13 Hz and 23 Hz, the curve of test results at 3 Hz is becoming unsmooth with the intensification of vibration excitation. That is because the shaker has a limited vibration moving space. When the root mean square value of vibration comes to 0.1g at 3 Hz, it is hard to continue to increase anymore. As a result, all the tests must be under a quite tiny vibration excitation, which directly leads to relatively low output power.

**
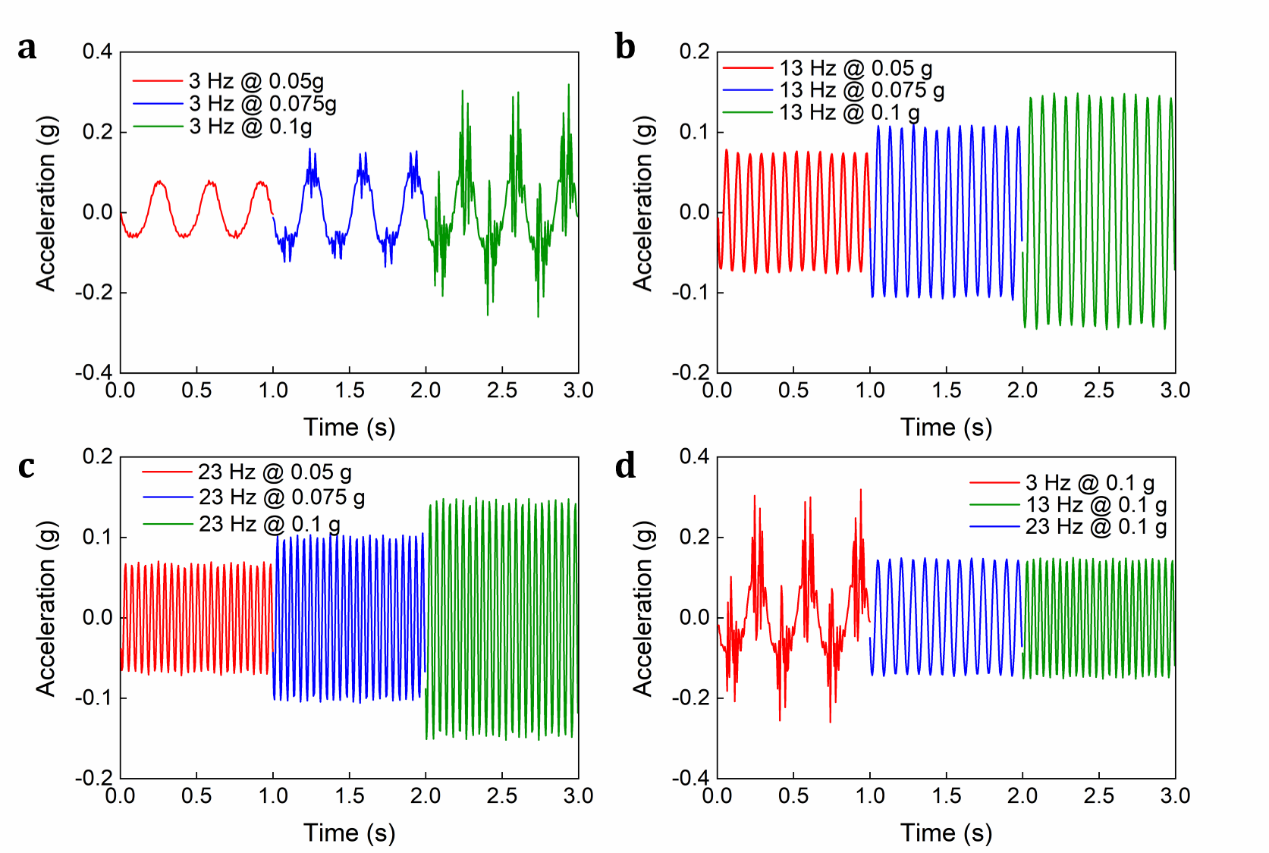
**

**Fig. S5 Acceleration tests for vibration excitation of different root mean square values. a** Vibration excitation @ 3 Hz with root mean square values of 0.05g, 0.075g, and 0.1 g. **b** Vibration excitation @ 13 Hz with root mean square values of 0.05g, 0.075g, and 0.1 g. **c** Vibration excitation @ 23 Hz with root mean square values of 0.05g, 0.075g, and 0.1 g. **d** Vibration excitation @ 3 Hz, 13Hz and 23 Hz respectively with root mean square value of 0.1 g.

**Reference**

S1. Sharpes, N., Abdelkefi, A. & Priya, S. Two-dimensional concentrated-stress low-frequency piezoelectric vibration energy harvesters. *Appl. Phys. Lett.* **107**, 093901 (2015).

S2. Park, J., Kim, H. S. & Han, A. Micropatterning of poly(dimethylsiloxane) using a photoresist lift-off technique for selective electrical insulation of microelectrode arrays. *J. Micromechanics Microengineering* **19**, 065016 (2009).
